# Exploiting Common Aspects of Obesity and Alzheimer’s Disease

**DOI:** 10.3389/fnhum.2020.602360

**Published:** 2020-12-15

**Authors:** Sidra Tabassum, Afzal Misrani, Li Yang

**Affiliations:** Precise Genome Engineering Center, School of Life Sciences, Guangzhou University, Guangzhou, China

**Keywords:** obesity, insulin resistance, neuroinflammation, Alzheimer’s disease, mitochondrial dysfunction

## Abstract

Alzheimer’s disease (AD) is an example of age-related dementia, and there are still no known preventive or curative measures for this disease. Obesity and associated metabolic changes are widely accepted as risk factors of age-related cognitive decline. Insulin is the prime mediator of metabolic homeostasis, which is impaired in obesity, and this impairment potentiates amyloid-β (Aβ) accumulation and the formation of neurofibrillary tangles (NFTs). Obesity is also linked with functional and morphological alterations in brain mitochondria leading to brain insulin resistance (IR) and memory deficits associated with AD. Also, increased peripheral inflammation and oxidative stress due to obesity are the main drivers that increase an individual’s susceptibility to cognitive deficits, thus doubling the risk of AD. This enhanced risk of AD is alarming in the context of a rapidly increasing global incidence of obesity and overweight in the general population. In this review, we summarize the risk factors that link obesity with AD and emphasize the point that the treatment and management of obesity may also provide a way to prevent AD.

## Introduction

The population of the developed world is aging, and the incidence of age-related metabolic and neurodegenerative diseases is increasing. Alzheimer’s disease (AD) is one of the most common age-associated neurodegenerative diseases; it is characterized by the accumulation of extracellular amyloid-β (Aβ) plaques and intracellular neurofibrillary tangles (NFTs; Jack, 2020; Sun et al., 2020). Mutations in one or more of the genes that encode amyloid precursor protein (APP), presenilin 1 (PS1), or presenilin 2 (PS2) represent genetic risk factors for AD (Gaiteri et al., 2016) and are typically associated with early-onset AD, but account for less than half of all cases. Conversely, late-onset AD is associated with environmental factors (more specifically, lifestyle; Hohman and Kaczorowski, 2020). Environmental risk factors that are associated with AD include vascular lesions, atherosclerosis, hypertension, glucose intolerance, insulin resistance (IR), hyperglycemia, hyperinsulinemia, appetite dysregulation, and obesity (Hayden, 2019). Despite these insights, there are still, after decades of research, no disease-modifying or preventive treatments available. Therefore, identifying modifiable risk factors and finding the mechanistic links with AD is of significant interest.

Obesity is currently one of the most widespread health threats to have reached epidemic proportions worldwide and is projected to reach 573 million cases by 2030 (World Health Organization, 2020). The excess fat (adipose) tissue that accumulates in the body due to a prolonged imbalance between calorie intake and expenditure can result in obesity (El-Mallah and Obeid, 2020). It has been reported that humans have elevated susceptibility to obesity and an increased risk of non-alcoholic fatty liver disease (NAFLD) and degenerative diseases compared to nonhuman primates (Martins, 2012). Aging-associated obesity causes decreased lean mass and increased risk of obesity-related diseases (Pugazhenthi et al., 2017). Moreover, the aging process may also link NAFLD, another chronic disease whose global epidemic is expected to reach between 20 and 40% by 2020, to obesity and AD (Bellentani et al., 2010). Consumption of a high-fat diet (HFD) is the prime cause of obesity and leads to various pathological conditions, including poor mental health, sleep apnea, and cognitive problems (Uranga and Keller, 2019). Although studies emphasize that obesity could increase the risk of dementia (Whitmer et al., 2007; Tezapsidis et al., 2009), the evidence is mixed for obesity as a risk factor for AD.

**Figure 1:**
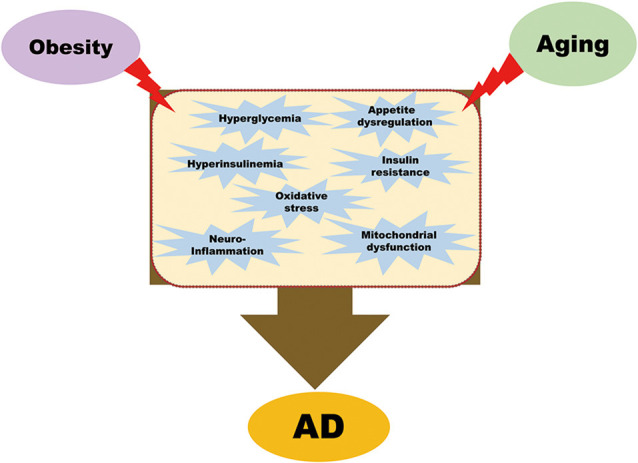
Common pathological mechanisms that link obesity with Alzheimer’s disease (AD). Obesity leads to hyperglycemia, hyperinsulinemia, insulin resistance, oxidative stress, appetite dysregulation, neuroinflammation, and mitochondrial dysfunction. All these pathological conditions, combined with aging, are contributory factors of AD.

The long-term effects of the obesity epidemic, coupled with an aging global population, are severe and burdensome. Because of the profound socio-economic impact of both AD and obesity, it is imperative to understand the mechanisms that could connect the two conditions. This review article aims to highlight the common factors linking obesity with AD; currently, these include insulin signaling pathways, oxidative stress, appetite dysregulation, neuroinflammation, and mitochondrial dysfunction in the brain, as summarized in Figure 1.

## Obesity and Progression of AD

According to the amyloid cascade hypothesis, AD-associated neurodegeneration is triggered by APP processing through the amyloidogenic pathway (Ow and Dunstan, 2014). The production of Aβ plaques is generally regarded as influencing neuronal activity by impairing synaptic function and inducing cell death (Hong et al., 2016). Concerning obesity and its influence on Aβ deposition, several studies have reported body weight gain in APP transgenic mice in response to high-calorie diets. Diets vary from high-fat and high cholesterol diets to a diet rich in sucrose. Strangely, given the variation in the nutrient content of the various regimens, diet-induced obesity is consistently linked to a rise in cerebral Aβ pathology (Levin-Allerhand et al., 2002; Fewlass et al., 2004; Ho et al., 2004; Cao et al., 2007; Pedrini et al., 2009; Julien et al., 2010; Shie et al., 2015). The only exception is a single study in which the authors examined the acute effects (i.e., after 4 weeks) of a “western” diet imposed on APP mice at 1−2 months of age (before Aβ deposition; Studzinski et al., 2009). Similarly, a strong association between weight loss and decreased levels of cerebral Aβ plaques has been reported using several dietary regimens including ketogenic and calorie-restricted diets (Patel et al., 2005; Van der Auwera et al., 2005; Wang et al., 2005; Halagappa et al., 2007; Mouton et al., 2009) in different APP transgenic strains, providing evidence that body weight, diet, and obesity are important modulators of Aβ pathology.

Another critical pathological feature of AD is NFTs, produced by hyperphosphorylated tau (Bartos et al., 2012). Multiple studies have demonstrated that tau pathology can be modulated by obesity (Leboucher et al., 2013; Platt et al., 2016). In a study to examine the dietary risk factors of AD, Julien et al. (2010) found that an HFD causes Aβ and tau pathologies in the frontal cortex of the 3xTg AD mouse model. The mitogen-activated protein kinase (p38, MAPK) signaling pathway is one of the major pathways disrupted in obesity (Katiyar and Meeran, 2007; Roth et al., 2009). This pathway is also linked with tau hyperphosphorylation and neuroinflammation-mediated NFT generation (Kelleher et al., 2007), and the association is further confirmed by the observation of increased tau hyperphosphorylation in hyperinsulinemic rats (Freude et al., 2005). Moreover, HFD-induced obesity in the murine model results in Aβ plaques, NFTs, and inflammation in the hippocampus (Puig et al., 2012).

Collectively, these data suggest that obesity influences Aβ aggregation and tau phosphorylation, promoting the pathogenesis of AD. Since insulin is an important mediator of obesity-related pathologies, we will focus in the next section on brain insulin and how elevated insulin levels may relate to neurodegenerative disorders.

## Brain Insulin Signaling and AD

Insulin mediates metabolic homeostasis by regulating glucose, lipids, and energy (Cheng et al., 2010b). The role of insulin in AD is currently of major interest (Arvanitakis et al., 2020; Selles et al., 2020). Insulin regulates glucose metabolism, both directly and indirectly. Insulin can be found in various brain areas under physiological conditions, especially in the cortex, hippocampus, and hypothalamus (Blazquez et al., 2014). The insulin levels in these brain regions are much higher than the insulin levels in plasma (Devaskar et al., 1994; Chiba et al., 2009; Kuwabara et al., 2011). Insulin is primarily derived from the blood and is transported to the brain *via* insulin binding sites present on brain endothelial cells (Hersom et al., 2018). Insulin receptors are widely present in various brain areas, especially those regions that regulate olfaction, cognition, appetite, and autonomic activity (Marks et al., 1991; Pomytkin et al., 2018). Of the glucose transporters (GLUT), GLUT-4 is particularly important for cellular glucose uptake in most peripheral tissues and is mainly regulated by insulin (Mourelatou et al., 2019). However, insulin fails to induce cellular glucose uptake into neurons, nor are insulin receptors activated, since insulin does not influence the translocation of GLUT-4 in the brain (Talbot et al., 2012). Accordingly, other types of glucose transporter, for example, GLUT-3, mediate neuronal glucose uptake in an insulin-independent fashion (Nagamatsu et al., 1994). Several studies have consistently shown that insulin is linked to cognitive functions in the brain rather than neuronal glucose uptake like in peripheral organs (Plum et al., 2005; Cholerton et al., 2013).

Increasing evidence indicates that insulin affects the brain in numerous ways: it exerts neuroprotective effects, works as a neuromodulator, and also plays a role in memory and cognition (Nampoothiri et al., 2017; Pitt et al., 2017). Various studies have shown that either peripheral or central insulin administration has beneficial effects on learning and memory (Lee et al., 2016; Pearson-Leary et al., 2018), which have been linked to insulin receptor activation and downstream signaling (Nelson et al., 2008; Lin et al., 2010; Chambers et al., 2015). Insulin receptor beta-subunit haploinsufficiency affects hippocampal late-phase long-term potentiation (LTP) and memory processing (Nistico et al., 2012), and also makes long-term depression (LTD) more likely (Huang et al., 2003; Ahmadian et al., 2004). In synaptic areas, insulin might also modulate the release of neurotransmitters, particularly glutamate, which is crucial for the maintenance of synaptic transmission (Ahmadian et al., 2004). In summary, these findings indicate that insulin plays an essential role in neuromodulation, neuroprotection, and cognition.

As intimated earlier, insulin can cross the blood-brain barrier (BBB) and competes with Aβ for the insulin-degrading enzyme (IDE) in the brain, including in the hippocampus (Farris et al., 2003). Besides, insulin is also produced in the brain, which may have a favorable effect on amyloid clearance (Reger et al., 2006). Hence, impaired or elevated levels of insulin could have detrimental effects on the brain. For example, impaired insulin signaling could increase, at least in part, Aβ accumulation and phosphorylation and cleavage of tau. As mentioned above, obesity is also a key contributor to metabolic dysfunction involving impaired insulin signaling, which leads to dysfunctional glucose metabolism. Hence, obesity-induced dysfunctional glucose metabolism might be the first common mechanism with relevance to AD.

## Glucose Metabolism in Obesity and AD

Glucose metabolism and insulin signaling are essential for the proper functioning of the brain. Increasing evidence suggests that hypometabolism of glucose might be a key player in dementia pathology (Kuehn, 2020). Remarkably, changes in glucose metabolism are also associated with AD since imaging studies typically show decreased glucose metabolism in the temporal and parietal brain regions of AD patients and individuals at risk of developing this disease (Small et al., 2000). Besides, patients with AD may also have elevated fasting plasma insulin levels, attenuated insulin and insulin-like growth factor (IGF) receptor expression, and reduced cerebrospinal fluid (CSF)-to-plasma insulin ratio relative to healthy individuals (Steen et al., 2005). Moreover, intravenous administration of insulin (while maintaining blood glucose levels) or glucose in AD patients and healthy older adults, improves cognitive function (Watson and Craft, 2004). These findings indicate that proper glucose metabolism is necessary for optimal cognitive function and that impairment of glucose metabolism leading to cognitive dysfunction is one of the characteristic features of AD.

### Insulin Resistance (IR)

IR is a pathological condition, often referred to as glucose intolerance, in which target tissues are not physiologically responsive to insulin. This may result in hyperinsulinemia occurring with euglycemia (Kim and Reaven, 2008). Hyperinsulinemia can interrupt the physiological function of several vital organs by impairing insulin signaling and disrupting intracellular signaling transduction (Zhang et al., 2007). Obesity is the major contributor to the induction of peripheral IR (Bacha et al., 2006; Jones et al., 2016; Fealy et al., 2018; Gao et al., 2019), resulting in overproduction of free fatty acids (FFAs) and causing oxidative stress (Tripathy et al., 2003). In rodents, chronic HFD-induced obese-IR exhibited a cognitive decline with impaired insulin regulation, increased inflammation, mitochondrial dysfunction, increased oxidative stress, and apoptosis in the brain (Porter et al., 2012; Sripetchwandee et al., 2014). These results suggest that chronic peripheral IR can induce brain IR and brain dysfunction.

IR also has relevance to AD because the metabolism of Aβ is mechanistically linked to IR. As we described earlier, insulin is important for amyloid clearance, while IR reduces the clearance of Aβ and facilitates its aggregation *via* sequestration of IDE (a key enzyme for Aβ degradation; Qiu and Folstein, 2006). Insulin also affects the production and removal of Aβ through the MAPK signaling pathway. In brief, hyperinsulinemia or IR induces MAPK signaling pathway activation and increases BACE1 expression, which eventually triggers the excessive accumulation of Aβ peptides and neuritic plaques. By contrast, IR inhibits the non-amyloidogenic pathway by reducing the expression of alpha-secretase and decreasing non-Aβ peptide production (Gasparini et al., 2001). Increased Aβ levels potentiate the removal of insulin receptors on the cell surface and further promote IR (De Felice et al., 2009). Although disrupted MAPK signaling is linked to the elevation of Aβ levels, it also results in the generation of NFTs facilitated by tau hyperphosphorylation and neuroinflammation by activating extracellular signal-related kinase (ERK) or direct phosphorylation of transcription factor such as cyclic AMP response element (CRE)-binding protein (CREB; Kelleher et al., 2007; Hu et al., 2012). Furthermore, IR causes decreased phosphoinositide 3-kinase (PI3K)/AKT pathway activation and facilitates activation of glycogen synthase kinase (GSK) 3β (one of the kinases involved in tau phosphorylation). Hence, increased GSK3β activation might cause hyperphosphorylation of tau and neurofibrillary lesions (Jolivalt et al., 2008). Additionally, IR blocks protein phosphatase 2A (PP2A) inhibition, which also leads to tau hyperphosphorylation and accumulation of NFTs (Gratuze et al., 2017).

**Figure 2:**
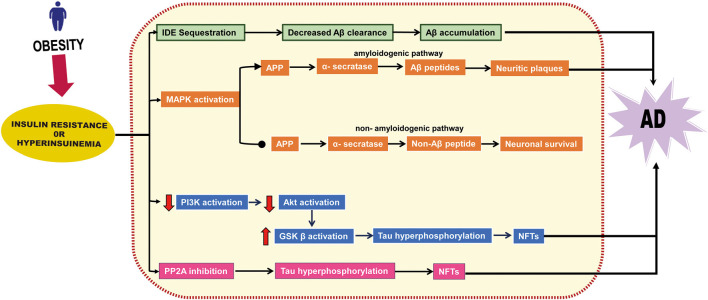
Insulin resistance or hyperinsulinemia-related neurodegenerative pathway. Insulin resistance or hyperinsulinemia induces degradation of insulin-degrading enzyme (IDE), affects the MAPK signaling pathway, stimulates the amyloidogenic pathway, and inhibits the non-amyloidogenic pathway, thereby facilitating the accumulation of amyloidβ (Aβ). It also induces Akt downregulation that eventually boosts glycogen synthase kinase (GSK) β activation and inhibits phosphatase 2A (PP2A) which leads to neurofibrillary tangles (NFTs) formation.

These studies demonstrate that obesity-induced IR and hyperinsulinemia cause brain dysfunction and facilitate NFT and Aβ accumulation, as summarized in Figure 2. Thus, obesity-induced IR or hyperinsulinemia represent a potential mechanistic link between obesity and AD.

### Advanced Glycation End Products (AGEs)

Advanced glycation end products (AGEs) are harmful compounds in the bloodstream when protein or fat combines with sugar during the glycation cycle (Yamagishi and Matsui, 2010). RAGEs are receptors of AGEs that seem to play a significant role in response to an HFD, both in terms of body mass regulation, macrophage content of adipose tissue, and systemic metabolism (Tomino et al., 2011; Leuner et al., 2012). Elevated AGE levels in both serum and tissues occur in an animal model of obesity (Li et al., 2005). The literature links AGEs to obesity *via* oxidative stress and inflammatory processes (Ramasamy et al., 2005). Nevertheless, it is not clear to what degree high levels of circulating AGEs are the cause or a symptom of obesity or how AGEs and RAGEs can be affected by body fat and lifestyle factors. Though, mechanistic insights into how AGEs cause oxidative damage indicate that this happens through RAGEs, which are expressed on a variety of cells, including endothelial cells, smooth muscle cells, fibroblasts, and neurons (Kalousova et al., 2005). Once an AGE binds a RAGE molecule, various signal transduction pathways, such as those involving NF-κβ or the MAPKs ERK and c-jun N-terminal kinase (JNK), can be activated. Consequently, gene transcription can be stimulated to increase the production of adhesion molecules, i.e., intercellular adhesion molecule-1 (ICAM-1), vascular cell adhesion molecule (VCAM-1) and growth factors like interleukin 1 (IL-1), IGF-1 and tumor necrosis factor (TNF-α). Moreover, it is also suggested that glycation can occur in DNA: AGE modification of DNA may thus have an effect on epigenetic regulation and other regulatory processes at the genetic level (Ramasamy et al., 2005). A low-AGE diet attenuates inflammatory profiles in humans (Harcourt et al., 2011), and an AGE inhibitor reduces glucose intolerance, hyperinsulinemia, loss of body weight, and fat deposition (Hagiwara et al., 2009). Earlier research focused on the effects of AGEs on visceral adipose tissue, but we suggest that ongoing and future work should probe whether and to what degree AGEs and expression of RAGEs influence brain function.

AGE formation also leads to oxidative damage, which is often referred to as glycoxidation (Rabbani et al., 2016). Glycoxidation is extremely relevant to AD, in part because extracellular fibrillar aggregates of Aβ have AGE characteristics and bind to RAGEs in neurons and endothelial cells of the brain. Glycation can delay Aβ conversion to fibrils, maintaining them in toxic oligomeric forms for longer (Emendato et al., 2018). Simultaneously, Aβ and AGE binding to RAGEs results in additional oxidative stress, which contributes to vascular dementia and neuronal death in AD (Takuma et al., 2009; Emendato et al., 2018; Wang et al., 2018). Furthermore, RAGEs mediate activation of GSK3β, which increases tau phosphorylation and cognitive decline (Li et al., 2012). Collectively, the above studies lead us to speculate that AGEs play a significant role in AD pathogenesis. However, how obesity-induced increased AGEs or RAGEs affect brain function and how they interact with AD remains a missing link.

## Neuroinflammation in Obesity and AD

**Figure 3:**
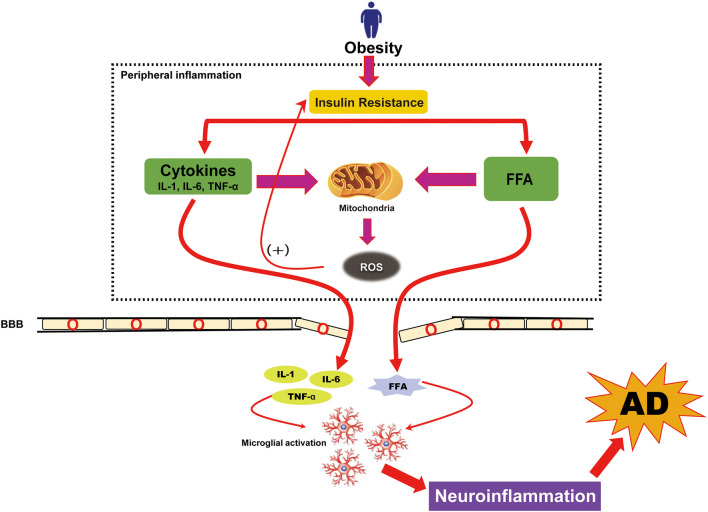
Obesity-induced neuroinflammation in AD. Neuroinflammation caused by obesity is known to arise from low-grade, chronic peripheral inflammation. Obesity-induced insulin resistance causes an overload of free fatty acids (FFAs) and triggers the activation of peripheral cytokines that further increased reactive oxidative stress (ROS). Although peripheral cytokines and FFAs can cross the blood-brain barrier (BBB), their overload causes damage to the BBB. The presence of increased FFAs and cytokines in the brain follows the cytokine production in brain cells (microglia) and causes neuroinflammation. This neuroinflammation is also a pathologic hallmark of AD.

Obesity is known to promote chronic low-grade systemic inflammation and neuroinflammation and is one of the most important mediators between obesity and AD (Gregor and Hotamisligil, 2011; Saltiel and Olefsky, 2017). Triglycerides (TGs), stored in the blood and adipose tissues, can be broken down into FFAs in individuals with obesity. FFAs initiate pro-inflammatory cytokine secretion from adipose tissue, which contributes to moderate and persistent systemic inflammation (Alford et al., 2018). Studies on obese and overweight adults showed altered levels of circulating inflammatory cytokines, including MCP-1, IL-6, IL-1β, and TNF-α, in these individuals (Chen et al., 2016). These altered levels of pro-inflammatory cytokines may establish an inflammatory milieu that reduces insulin sensitivity through feedback inhibition of the insulin receptor. This also disrupts mitochondrial function through a feed-forward mechanism, which then stimulates the production of reactive oxygen species (ROS) further to promote inflammation (Bonnard et al., 2008; Hoeks and Schrauwen, 2012). Such a chronic inflammatory milieu may stimulate NFκ-B-inducing kinase activity, which independently promotes further IR by compromising mitochondrial function (Figure 3; Choudhary et al., 2011). These cytokines can cross the BBB and facilitate the extravasation of leukocytes from the circulation through the BBB into the central nervous system (CNS). Also, chronic inflammation causes damage to the BBB that can have deleterious effects on the CNS, including hypothalamic dysfunction, loss of synapses, impaired cognition, and neurodegeneration (Wyss-Coray and Mucke, 2002; Gregor and Hotamisligil, 2011; Bettcher and Kramer, 2013). The inflammation caused by obesity can, therefore, result in neuronal damage, usually beginning in adolescence.

While there is a minimal passage of FFAs across the BBB, positron emission tomography has demonstrated fatty acid uptake in the brain of obese individuals (Karmi et al., 2010). The presence of carnitine in various brain regions indicates that FFAs are involved in brain metabolism (Mitchell and Hatch, 2011). Nevertheless, toxic-level accumulation of fats in the brain can lead to damaging effects, such as inflammation of the brain. Long-chain fatty acids in the brain function *via* TLRs such as TLR4 and trigger cytokine production in local cells (microglia), eventually leading to activation of NF-κB signaling (Gupta et al., 2012). Hence, persistent CNS inflammation causes cerebral IR, hyperinsulinemia, and hyperglycemia and consequently follow the production and deposition of AGE (Alford et al., 2018). Nevertheless, obesity-induced chronic low-grade inflammation in midlife provides a mechanistic association with a progressive cognitive decline as a result of CNS inflammation (Pugazhenthi et al., 2017).

Neuroinflammation is also considered a major contributor to AD pathogenesis. The pathological accumulation of Aβ alone may be sufficient to induce an inflammatory environment, which consequently contributes to progressive cognitive decline and AD (Guerriero et al., 2017). Considering the likelihood of Aβ deposition preceding cognitive impairment or clinical manifestation, one might speculate that exogenous or endogenous factors may alter the innate immune response of microglia exposed to Aβ. We have summarized the molecular pathway in which environmentally modifiable AD risk factors such as obesity might affect AD pathogenesis through a sustained neuroinflammatory drive (Figure 3).

## Mitochondrial Dysfunction in Obesity and AD

Mitochondria are usually referred to as the cellular powerhouses as they provide energy to the cell (Morrow et al., 2017; Bertolin et al., 2018). Mitochondrial dysfunction has been observed in both AD and obese individuals with insulin resistance (Vernochet and Kahn, 2012; Ji et al., 2015; Swerdlow, 2020). As well as adenosine triphosphate (ATP) production, mitochondria play numerous roles in neurons such as Ca^2+^ regulation, lipid metabolism, ROS signaling, and cell survival or death (Torralba et al., 2016; Pfanner et al., 2019). Regarding brain activity, studies have shown that mitochondria are important for cognitive function and synaptic transmission (Mattson et al., 2008; Cheng et al., 2010a; Raefsky and Mattson, 2017; Lee et al., 2018). Specifically, presynaptic mitochondria endorse sustained synaptic activity by providing ATP and buffering presynaptic Ca^2+^ signals, thereby modulating neurotransmission and eventually imposing an upper limit on synaptic activity. Also, mitochondrial morphological changes in presynaptic neurons impair synaptic homeostasis, and may, therefore, lead to neurodegeneration (Devine and Kittler, 2018). Morphologically, donut-shaped mitochondria are indicative of mitochondrial stress (Liu and Hajnoczky, 2011; Ahmad et al., 2013) and correlate with the deterioration of working memory with aging (Hara et al., 2014). In subsequent studies, the authors found that donut mitochondria are linked with reduced synapse formation, as demonstrated by smaller active zone sizes. Moreover, donut-containing presynaptic terminals have fewer fully docked vesicles, which indicates a reduced potential for the release of synaptic vesicles containing neurotransmitters. Hence brain mitochondria, and more specifically, presynaptic mitochondria, significantly affect cognitive function (Dragicevic et al., 2010; Baek et al., 2017), which supports the hypothesis that mitochondrial dysfunction may result in cognitive impairment. Similarly, mitochondrial Aβ accumulation and mitochondrial dysfunction were observed in an AD mouse model, and the degree of these impairments correlated with the extent of cognitive decline (Dragicevic et al., 2010).

Brain mitochondrial dysfunction is also linked with an obese/insulin-resistant condition (Hunnicut et al., 2015; Koliaki and Roden, 2016; Thoudam et al., 2019). Both an HFD and genetically mediated obesity/IR consistently cause mitochondrial dysfunction characterized by alterations in mitochondrial membrane potential, excessive mitochondrial ROS production, and swollen mitochondria with unfolded cristae (Ciapaite et al., 2007; Cardoso et al., 2010; Gomes et al., 2012; Guo et al., 2013; Kalinovich et al., 2016). A reduction in ATP levels as a consequence of mitochondrial dysfunction, i.e., reduced O_2_ consumption and excessive CO_2_ output, occurs in obese/IR rats (Porter et al., 2012, 2013; Raza et al., 2015; Wang et al., 2015). These studies only reported on the association between obesity/IR and dysfunction of brain mitochondria and brain IR (Pintana et al., 2013; Pipatpiboon et al., 2013; Sa-Nguanmoo et al., 2016, 2017), but to date, it is still unclear how peripheral mitochondrial dysfunction leads to brain IR and brain mitochondrial dysfunction.

Mitophagy, a type of autophagy, plays a significant role in maintaining a healthy mitochondrial pool and ensuring neuronal function and survival, and mitophagy deficits might be the leading pathological cause of Aβ enrichment (Wang et al., 2016). Pink-1-parkin and Sirtuin (Sirt) mediated mitophagy pathways are getting more focus with relevance to chronic diseases, including AD (Fang et al., 2016) and obesity (Ren et al., 2020). Sirt-1, a member of the deacetylase protein family exerting protective effects against cellular insult (Zhang et al., 2016), is involved in the regulation of a variety of cellular stress responses such as inflammation, autophagy, and apoptosis (programmed cell death; Hall et al., 2013). Some studies link brain apoptosis with mitochondrial dysfunction (Sa-Nguanmoo et al., 2016, 2017). One possible explanation is that, due to mitochondrial swelling, cytochrome c is released, resulting in the development of a complex with apoptotic protease activating factor-1 (APAF-1). These complexes become the apoptosomes and activate caspase cascades, which eventually trigger apoptosis (Cozzolino et al., 2006; Spellicy et al., 2018). In line with the above notions, it was found that mitochondrial dysfunction causes enhancement of pro-apoptotic protein (Bax and Bad) levels, and reduction in anti-apoptotic protein (Bcl-2) levels in the brain of obese/IR rats (Nuzzo et al., 2015; Sa-Nguanmoo et al., 2016, 2017). An increase in pro-apoptotic proteins can cause the release of cytochrome c, leading to apoptosis in the brain (Gomez-Lazaro et al., 2007; Gomez-Crisostomo et al., 2013). Apoptosis-mediated neuronal death has also been observed in several neurodegenerative diseases and is considered a key process underlying cognitive dysfunction (Ghavami et al., 2014).

**Figure 4:**
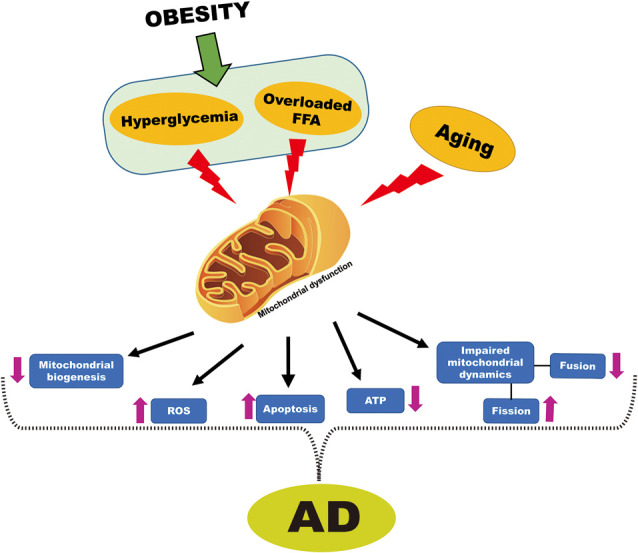
Obesity-induced mitochondrial dysfunction cross-talk with AD. Hyperglycemia and overloaded FFA that are associated with obesity and aging are known to cause mitochondrial dysfunction. Dysfunctional mitochondria decreased mitochondrial biogenesis, increased ROS, increased apoptosis, reduced adenosine triphosphate (ATP) generation, impaired mitochondrial dynamics, which is characterized by increased fission and reduced fusion. All of these events are also associated with mitochondrial dysfunction in AD.

Besides the generation of energy and apoptosis, mitochondrial processes such as fission and fusion also support cell survival or death (Perfettini et al., 2005). Neurodegeneration and cognitive dysfunction are associated with an imbalance in mitochondrial dynamics as well as with brain mitochondrial dysfunction (Knott and Bossy-Wetzel, 2008; Bertholet et al., 2016). Previously, it was reported that the imbalance in mitochondrial dynamics implied by a reduction in mitochondrial fusion in conjunction with an increase in mitochondrial fission, leads to cognitive dysfunction and cell death (Cho et al., 2010). More specifically, mitochondrial fission protein (Drp-1) is involved in synaptic dysfunction and neurodegeneration as Drp-1 inhibitor improved age-related synaptic depression, cognitive decline, and Aβ accumulation in APP/PS1 mice (Baek et al., 2017). Similarly, this imbalance in mitochondrial dynamics is also seen in obesity/IR, as shown by decreased expression of mitochondrial fusion proteins and increased expression of mitochondrial fission proteins (Diaz et al., 2015; Filippi et al., 2017). Hence the underlying mechanisms responsible for age-related cognitive decline or cognitive dysfunction in obesity/IR are likely either imbalanced mitochondrial dynamics or impaired brain mitochondrial function. Based on the above reports, there is strong evidence to suggest that obesity leads, at least in part, to the onset of AD by compromising mitochondrial function (Figure 4).

## Appetite Dysregulation in Obesity and AD

Dietary/ appetite regulation of the nuclear receptors involves the Sirt-1 gene (also known as calorie sensitive anti-aging gene; Martins, 2016b), which has a close link with the development of AD (Pardo and Boriek, 2020). In global populations, the AD-associated appetite dysregulation in early life shows defective suprachiasmatic nucleus (SCN) in the appetite center, hypothalamus (Reisberg et al., 1989; Nifli, 2018). Physiologically, Sirt 1 is involved in Aβ clearance from neurons and inhibiting inflammatory responses in neuroglia, thus functions as a neuroprotective factor against Aβ toxicity and cognitive deficits (Li et al., 2018). Conversely, during the aging process, Sirt-1 activity is suppressed due to transcriptional dysregulation, leading to defective mitophagy and AD accelerations (Yuan et al., 2016). Additionally, Sirt-1 also plays a significant role in the glucose and fat homeostasis by regulating various transcription factors; reduction in Sirt-1 activity due to either HFD or unhealthy diet leads to insulin resistance (Chen et al., 2013), altered immunity, mitochondrial apoptosis, and NAFLD (Martins, 2017b). Moreover, Nitric oxide (NO) is one of the principal regulators of various mitochondrial functions such as mitochondrial biogenesis and mitochondrial respiration (Nisoli et al., 2003; Bombicino et al., 2017) and appetite regulation (Morley et al., 2011); whose disturbances are extensively reported in various chronic diseases, such as AD (Tse, 2017) and obesity (Rus et al., 2016). Existing literature reported that Sirt-1 prevents oxidative stress by maintaining NO levels and creating ROS resistance (Zhang et al., 2017). These reports, together, suggest that appetite dysregulation may be critically involved in obesity and AD pathogenesis.

## Discussion and Conclusion

While the mechanisms underlying AD are not yet completely understood, it is evident that AD follows a series of critical events in the brain over time. There is convincing evidence that obesity is linked with AD, especially in middle age and in younger adults. This association alone makes understanding the mechanisms and pitfalls addressed in this review essential. The connections between dementia, obesity, and aging represent a major threat to public health, particularly as people are adopting increasingly sedentary lifestyles. The preference is for a western-style diet with high levels of starch, fat, sugar, and oil, and this is coupled with reduced energy expenditure. This “double whammy” creates an imbalance between calorie intake and expenditure that collectively leads to obesity. Though the severity of obesity and its relevance to AD differs between developing and developed countries (Martins, 2013), the quality of food and xenobiotics levels might underlie this discrepancy (Martins, 2016a). A report on childhood obesity predicts that, by 2030, over 250 million young people will be classified as obese, from school-aged children to adolescents, thereby placing a massive burden on health care systems. While there have been numerous attempts to eliminate the causes and consequences of obesity, the management of blood glucose combined with weight loss seems to be, currently, the only successful strategy. However, the therapeutic interventions to prevent these interlinked diseases are still obscure.

Accordingly to the current literature, the most likely mechanisms linking obesity to AD involve IR, AGE accumulation, neuroinflammation, mitochondrial dysfunction, and appetite dysregulation. Though, oxidative stress and hyperglycemia contribute to AD pathogenesis as they potentiate Aβ deposition, hyperphosphorylation of tau, and eventually neuronal and synaptic failure. Hyperinsulinemia or IR, and their role in AD progression, have fostered tremendous interest recently and are thus topics of intensive research. However, it is still unclear whether these are just correlations or whether there is a direct effect on the amyloid cascade and AD, and whether any intervention will modify the risk of disease. In our opinion, this is the most important question in the field.

As discussed earlier, obesity-associated inflammation facilitates AD; hence neuroinflammation might be a critical pathway that accelerates the aging process, and its malfunction leads to alteration in other intersecting pathways related to obesity and AD as discussed in this review. Indeed, chronic neuroinflammation contributes to the development and progression of AD, but whether peripheral inflammation triggers neuroinflammation in AD is not entirely clear. More work is therefore required to identify the origin of inflammation in AD. Additionally, the role of TNF-α and NF-kB upregulation in neurons is extremely complex, and it is difficult to define any clear relationship with AD onset; further studies are therefore necessary for obese brain tissue. Although studies also correlate AD with mitochondrial dysfunction in obesity, how peripheral mitochondrial dysfunction causes dysfunctional brain mitochondria, and how this leads to AD is still unknown. We propose that the brain mitochondrial involvement in early pathogenesis should receive more emphasis on ongoing AD research programs. Based on what is understood about the natural history of obesity and our analysis of current evidence, we believe that obesity, when combined with aging, plays a significant role in AD pathogenesis.

One of the major risk factors of obesity is an unhealthy diet, which is involved in epigenetic modifications that further affect nuclear/mitochondria interactions and inactivate Sirt-1, thus accelerating NAFLD and obesity in the global population (Martins et al., 2015; Pardo and Boriek, 2020). Since repression of Sirt-1 is involved in obesity and AD, proteomic profiles that include early plasma Sirt-1 detection could be used as a biomarker associating with the inactivation of rapid toxic Aβ and therapeutic drug metabolism (Martins, 2018). Hence, a diagnostic test that can detect Sirt-1 inactivation, will provide an early insight into later disease alterations. Furthermore, as early nutritional intervention causes activation of the Sirt-1 pathway that reverse NAFLD with reduced obesity and prevent mitochondrial apoptosis (Martins, 2017a); therefore, pharmacological interventions which either prevent downregulation of Sirt-1 or upregulation of Sirt-1 would be beneficial for people with obesity and AD.

Obesity is contributing to glucose sensitivity and hyperinsulinemia/IR, which are known AD risk factors. IR is responsible for tau hyperphosphorylation and Aβ aggregation *via* the various mechanisms discussed in this review, leading to the pathogenesis of AD. All these factors can occur sequentially, but may also coexist. It is currently unknown how early lifelong obesity affects the risk of AD, but given the impact of hyperinsulinemia on brain amyloid metabolism the onset of the conditions that underlie the disease could begin at a young age or even in childhood. Since there is an increasing trend of obesity among children and adults, this possibility is overwhelming. Thus, it will be important to use traditional measures of obesity such as BMI in younger persons, and to study the correlation of obesity and AD, to attempt to clarify conflicting findings in the literature. Ethnicity and gender may also have differential effects on obesity, so we suggest taking ethnic group, age, and gender into consideration irrespective of the cut-offs for these measures. Additionally, it will be important to directly measure body composition in older adults to better understand the effects of obesity in this key subset of the population. Further research needs to determine how the prevention, treatment, and lifelong care of obesity might delay or reduce the risk of AD.

## Author Contributions

ST and LY designed the theme of the manuscript. ST wrote most sections. AM wrote one section of the manuscript. All authors reviewed the manuscript before submission. All authors contributed to the article and approved the submitted version.

## Conflict of Interest

The authors declare that the research was conducted in the absence of any commercial or financial relationships that could be construed as a potential conflict of interest.
